# Loot

**Published:** 1869-04-01

**Authors:** 


### LOOT.

The one hundred thousand destitute children of London are quaintly, and not inappropriately, designated by a cotemporary, *crime seed.*

### *Pepsine.*

The *Chemical News* makes a statement in reference to this product, which contains a valuable suggestion to Western manufacturers, viz.: That the strongest pepsine is obtained from young pigs, which, after having been kept hungry, and then having the gastric secretion excited by savory food, are pithed. The above suggests a means of obviating the greatest objection to the extended use of this article, viz., its high price.

***Pigeons and Opium.***

Dr. S. W. Mitchell (*Am. Jour. Med. Sciences*) having administered this drug to pigeons, in various forms and in quantities gradually increased to the enormous amount of twenty-six grains, has demonstrated their almost entire unsusceptibility to its influence.

Dr. Salisbury, of Cleveland, Ohio (*St. Louis Med. Reporter*), in pursuit of his investigations into the realm of the infinitesimal, has discovered a new cause for admiration at the wisdom of Moses in his prohibitory regulations against the hog, viz.: That we are indebted to that useful (?) animal for another boon in addition to Trichina, Tapeworm, etc. The Steatozoon Folliculorum, which the Doctor asserts has its original and natural habitation in the skin and surface adipose of the hog, and is then transmitted to the folliclee of the "human face divine."

***Holt's Operation for Stricture.***

*The Medical Press and Circular* reports a case of death following this operation, attributed to systemic infection, the corpus cavernosum having been lacerated, and containing pus.

***Medicated Pessaries.***

Dr. Meadows has proposed to the London Obstetrical Society the substitution of soft soap made into mass with powdered althea, starch or beeswax, for the cocoa butter usually employed in the formation of medicated pessaries. The advantages claimed being greater cleanliness and certainty in action.

The inability of the vagina to absorb fat, occasioning a probable loss of the medicinal ingredients, which being protected by a covering of fat, are passed away without being absorbed.

***Syr. Ferri Iodid.***

A writer in the *London Pharmaceutical Journal* states that he has in his possession a bottle of Syr. Ferri Iodid., which has been made more than twelve months; it is simply corked, and has been frequently exposed to the air. During the last six months the bottle has been kept only half-full, and the syrup is still in perfect preservation. The method adopted to secure this result is the simplest conceivable; it consists in exposing the syrup to sunlight for half an hour daily.

***Styptic Paper.***

It is well known that the perchloride, or as it is some times called, the muriate of iron, possesses styptic or hæmostatic properties in a high dregree. To obviate the inconvenience of carrying it about in solution, and also of applying it, M. Gustave Gabillon, of Paris, has devised a "styptic paper," the method of preparing which is as follows:

In a vessel carefully tinned inside, he keeps boiling for four hours (skimming from time to time, and adding water to supply that evaporated) a solution of one pound of gum benzoin, one pound of rock alum and four and a third gallons of water. As soon as the solution is cooled it is to be filtered and the paper or tissue to be dipped into it, kept there until saturated, and then carefully dried, after which a solution of the perchloride, more or less concentrated, is applied with a brush or a roller. The paper or tissue thus prepared may be preserved for any length of time in a state always ready for use, by excluding it from air.—*Druggist Circular and Chemical Gazette.*

***Gonorrhœ.***

Dr. C. P. Judkins reports to the Cincinnati *Lancet*, three cases of gonorrhœ, aborted after the lapse of one week, in each case, by large doses (one-half to three-quarters of a drachm) of pure copaiba, from three to four times daily, before meals. One of the cases was complicated with cystitis. The Doctor objects to the use of injections *in toto*, as applied to this disease, asserting that the inflammation is perpetuated by the irritation of the syringe.

***Chlorea.***

Dr. Willis (Guy's Hospital), discussing this disease doubts that this malady is due to any special disease of the nervous system, but rather like epilepsy, to a disturbance of the whole of the centres; that a weakened condition of the nervous centres being at the root of the malady good nourishment and tonics are indicated, and that medicines having a physiological action upon the nervous system have far less efficacy in controlling this disease than the metallic tonics. —*Boston Med. and Surg. Journal.*

### *Poisonous Red Dyes.*

M. Tardieu reports two cases of cutaneous disease occasioned by red dye in stockings. This dye, when extracted by a skillful chemist, by means of boiling water and alcohol, was injected under the skin of a frog, a rabbit and a dog. These animals all died after periods of four, thirty-six and forty-eight hours respectively, exhibiting the following post mortem appearances: violent inflammation, and purulent infiltration of the cellular tissue at the point of injection; intestines inflamed and distended with liquid fæces, liver involved in fatty degeneration, and lungs uniformly tinted with coloring matter. This substance was extracted from the lungs and livers of these animals and used to dye a skein of silk.

M. Tardieu compares the action of this dye (coralline) to that of croton oil.—*Boston Med. and Surg. Journal.*

### *Antidote for Strichnia.*

J. Dabney Palmer, in a communication to the *Am. Jour. of Pharmacy*, reports the poisoning of four cats by means of strychnia; to two of them the alkaloid was administered on pieces of beef; both died. To the other two it was given on small tufts of valerian, without the least effect. He asks, "Is valerian an antidote for strychnia?"

### *Tendo Achillis.*

M. Delore, of la Charité Hospital, Lyons, reports to the *Bulletin de Thèrapie* the case of a boy, nine years of age, who had severed his tendo achillis with a hatchet. Twelve days after the accident the tendon was exposed, and certain adhesions having been separated, the cut edges were pared and coaptated by four metallic sutures. No traction was permitted upon the sutures, which was prevented by position of the leg. Union had not yet taken place after the lapse of from ten to fourteen days; when the sutures were removed. No separation had occurred; the starch bandage was retained until the fifty-second day. Mobility of the limb, impaired by long confinement, was completely restored in six weeks, at which time perfect use of limb was recovered.—*Boston Med. and Surg. Journal.*

---

Dr. Samuel J. Jones, late Surgeon in the U. S. Navy, has been appointed Oculist and Aurist to St. Luke's Hospital.

